# Attenuating persistent sodium current–induced atrial myopathy and fibrillation by preventing mitochondrial oxidative stress

**DOI:** 10.1172/jci.insight.147371

**Published:** 2021-10-28

**Authors:** Uma Mahesh R. Avula, Haikel Dridi, Bi-xing Chen, Qi Yuan, Alexander N. Katchman, Steven R. Reiken, Amar D. Desai, Samantha Parsons, Haajra Baksh, Elaine Ma, Parmanand Dasrat, Ruiping Ji, Yejun Lin, Christine Sison, W. Jonathan Lederer, Humberto C. Joca, Christopher W. Ward, Maura Greiser, Andrew R. Marks, Steven O. Marx, Elaine Y. Wan

**Affiliations:** 1 Division of Cardiology, Department of Medicine, and; 2 Department of Physiology and Cellular Biophysics and Clyde & Helen Wu Center for Molecular Cardiology, Vagelos College of Physicians and Surgeons, Columbia University, New York, New York, USA.; 3 Center for Biomedical Engineering and Technology and Department of Physiology and; 4 Department of Orthopaedics, University of Maryland School of Medicine, Baltimore, Maryland, USA.; 5 Department of Molecular Pharmacology and Therapeutics, Vagelos College of Physicians and Surgeons, Columbia University, New York, New York, USA.

**Keywords:** Cardiology, Arrhythmias, Cardiovascular disease, Sodium channels

## Abstract

Mechanistically driven therapies for atrial fibrillation (AF), the most common cardiac arrhythmia, are urgently needed, the development of which requires improved understanding of the cellular signaling pathways that facilitate the structural and electrophysiological remodeling that occurs in the atria. Similar to humans, increased persistent Na^+^ current leads to the development of an atrial myopathy and spontaneous and long-lasting episodes of AF in mice. How increased persistent Na^+^ current causes both structural and electrophysiological remodeling in the atria is unknown. We crossbred mice expressing human F1759A-Na_V_1.5 channels with mice expressing human mitochondrial catalase (mCAT). Increased expression of mCAT attenuated mitochondrial and cellular reactive oxygen species (ROS) and the structural remodeling that was induced by persistent F1759A-Na^+^ current. Despite the heterogeneously prolonged atrial action potential, which was unaffected by the reduction in ROS, the incidences of spontaneous AF, pacing-induced after-depolarizations, and AF were substantially reduced. Expression of mCAT markedly reduced persistent Na^+^ current–induced ryanodine receptor oxidation and dysfunction. In summary, increased persistent Na^+^ current in atrial cardiomyocytes, which is observed in patients with AF, induced atrial enlargement, fibrosis, mitochondrial dysmorphology, early after-depolarizations, and AF, all of which can be attenuated by resolving mitochondrial oxidative stress.

## Graphical abstract

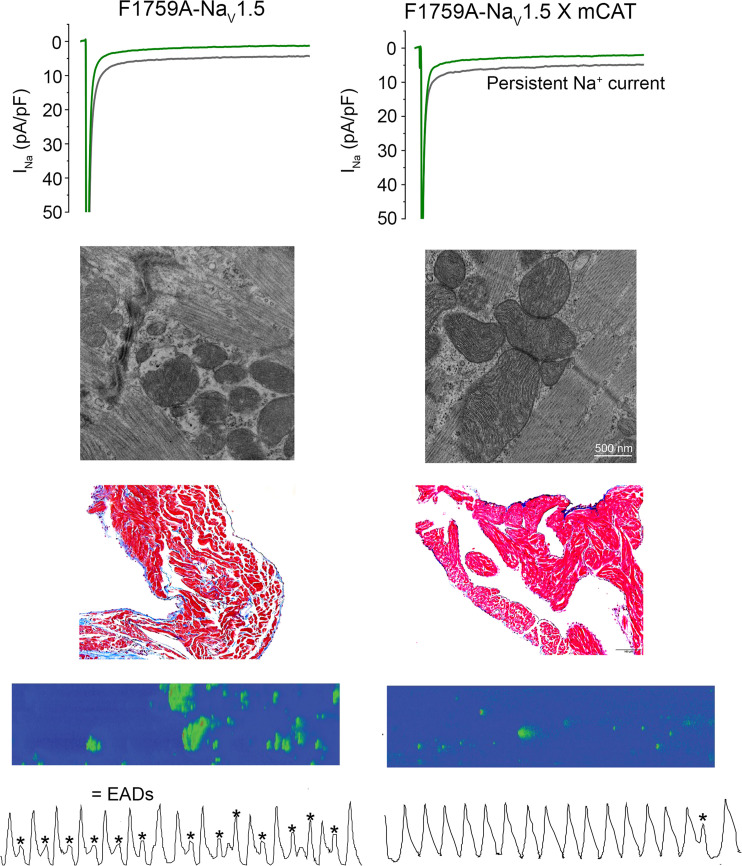

## Introduction

The incidence of atrial fibrillation (AF) increases with age, affecting 1% of individuals 60–65 years old and 8%–10% of individuals older than 80 years (1, 2). AF treatments, in general, have suboptimal efficacy, toxicities, and high rates of recurrences. These challenges persist because many of the therapies, both pharmacological and ablative, are not directed at the underlying atrial myopathy, focusing instead on modifying the properties of the action potential or conduction.

In most patients with AF, the predisposing contributors to AF are systemic and cardiac disorders, including hypertension, heart failure, and valvular disease, which may ultimately lead to atrial enlargement, fibrosis, and electrical abnormalities (3). In rarer situations, AF can be caused primarily by an electrical disorder (“lone AF”) or via a genetic susceptibility, shown by recent genome-wide association studies and identification of relatively rare mutants in cardiac potassium (K^+^) and sodium (Na^+^) channels and ryanodine receptors (RyR2) (4–7). AF caused by primary electrical abnormalities and the more commonly occurring AF, secondary to systemic factors, may share at least some common mechanisms.

Mechanistic studies of AF have been hindered by the lack of a mouse model that accurately recapitulates the spontaneous initiation and prolonged periods of AF observed in humans. Most studies with mice use nonphysiological methods, including burst pacing, to induce short episodes of AF in mice (8, 9). In contrast, expression in mice of a gain-of-function mutant Na_V_1.5 channel causing increased persistent Na^+^ current leads to the development of an atrial myopathy and spontaneous and long-lasting episodes of AF (10). The atrial myopathy is notable for atrial dilatation, atrial fibrosis, and ultrastructural abnormalities such as myofibril disarray and mitochondrial injury (10). The increased persistent Na^+^ currents in this mouse line mimic observations in humans: gain-of-function sodium voltage-gated channel alpha subunit 5 (*SCN5A*) genetic variants are associated with patients with lone AF (11), an increased incidence of AF in patients with congenital long QT syndrome type 3 (12), and an increased persistent Na^+^ current in patients with permanent AF (13). Moreover, heart failure, hypoxia, inflammation, oxidative stress, and pharmacological agents can either delay or destabilize Na^+^ channel inactivation, thereby increasing persistent current (14), implying that the mechanism has more general applicability. We found that the heterogeneity of the electrophysiological substrate, namely variable expression of the mutant Na_V_1.5 channels leading to gradients of action potential duration (APD), was required for rotational reentry (15). Homogenizing the electrophysiological substrate, either by shortening or by prolonging the APD, markedly attenuated the initiation and perpetuation of AF, despite the presence of early after-depolarizations.

We sought to gain insights into the mechanisms underlying the persistent Na^+^ current-induced changes in the atrial substrate, including atrial enlargement, fibrosis, and the generation of early after-depolarizations (EADs). We reasoned that increased oxidative stress could be a critical factor in mediating these Na^+^ current-induced changes in cardiac function. Oxidative stress, an imbalance between the generation and neutralization of ROS, is believed to be one mechanism through which AF is initiated and sustained (16). In the atrial appendages of patients with AF, increased inflammation and oxidative injury was found compared with those patients in normal sinus rhythm. NADPH oxidase (NOX2) is a major enzymatic source of ROS in the fibrillating atrium (17). Not only does increased ROS in cardiomyocytes alter electrophysiological properties of atrial ion channels, including Na_V_1.5 channels (18) and RyR2 (19), thereby promoting arrhythmias, but it also leads to activation of genetic programs, of atrial myofibroblasts, and of matrix metalloproteinases that ultimately result in cardiac hypertrophy (20). Additionally, activated atrial myofibroblasts secrete extracellular matrix and, through the production of cytokines and chemokines, trigger an inflammatory response. Risk factors for AF, such as hypertension, diabetes, and advanced age, are associated with increased ROS (21–23).

Here, we show that increased mitochondrial ROS drives changes in the atrial substrate that underlie the development of spontaneous AF induced by increased persistent Na^+^ current. Attenuating mitochondrial ROS by expressing mitochondrially targeted catalase reduced atrial dilatation and fibrosis and spontaneous AF in the transgenic mice expressing mutant Na_V_1.5 channels. The reduction in spontaneous and burst pacing–induced AF was likely secondary to several factors, including decreased frequency of after-depolarizations, reduced oxidation of RyR2 channels and diastolic Ca^2+^ release, and reduced fibrosis and atrial dilatation, despite continued heterogeneously prolonged APD caused by persistent Na^+^ current. Taken together, targeting mitochondrial ROS may protect against persistent Na^+^ current-induced cardiotoxicity.

## Results

**Figure 1:**
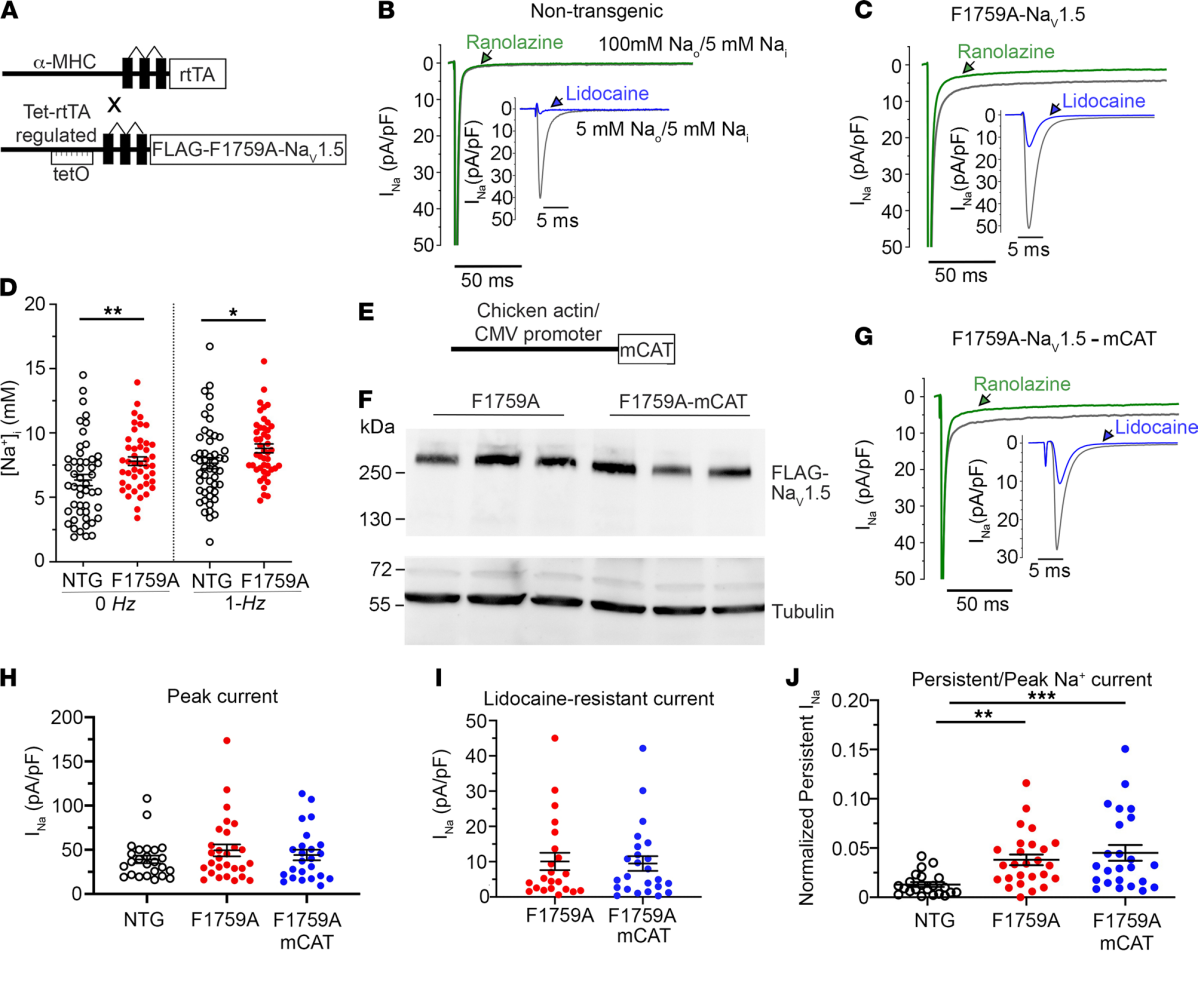
Expression of mitochondrial catalase does not attenuate F1759A-induced Na^+^ current. (**A**) The transgene system permitting expression of FLAG-F1759A-Na_V_1.5 when reverse tetracycline-controlled transactivator (rtTA) and doxycycline are present (Tet-ON). Top: rtTA-driven expression by the cardiac-specific α–myosin heavy chain (α-MHC) promoter. The 3 noncoding exons that make up the 5′-UTR of the α-MHC gene are depicted as boxes and the introns as lines. Bottom: cDNA for FLAG-F1759A-Na_V_1.5 ligated behind 7 tandem *tetO* sequences. (**B**, **C**, and **G**) Exemplary whole-cell Na^+^ current (I_Na_) traces of atrial cardiomyocytes isolated from control nontransgenic, F1759A-Na_V_1.5, and F1759A-mCAT mice. Persistent I_Na_ was evaluated with a 190 ms depolarization from a holding potential of –110 mV to –30 mV in the absence (black) and presence (green) of ranolazine; 5 mM Na^+^ in the intracellular solution, and 100 mM Na^+^ in the extracellular solution. Inset: Peak I_Na_ and fraction of lidocaine-resistant current, whole-cell current traces were recorded with 5 mM Na^+^ in extracellular and intracellular solutions, in the absence (black) and presence (blue) of 3 mM lidocaine. (**D**) Intracellular Na^+^ concentration ([Na^+^]_i_) in nontransgenic (NTG) and F1759A-Na_V_1.5 in quiescent (0 *Hz*) and field-stimulated (1 *Hz*) atrial cardiomyocytes. Two-way repeated measures ANOVA, *P* = 0.016, Tukey’s multiple-comparison test: * *P* < 0.05, ** *P* = 0.01. *n* = 5 mice/group. (**E**) Mitochondria-directed catalase–driven expression by chicken actin/CMV promoter (28). (**F**) Anti-FLAG (upper) and anti-tubulin immunoblots (lower) of cardiac homogenates of F1759A-Na_V_1.5 and F1759A-mCAT mice. Representative of 3 experiments. See complete unedited blots in the supplemental material. (**H**) Peak I_Na_ density recorded with 5 mM external Na^+^. *P* = 0.46; 1-way ANOVA. (**I**) Capacitance-normalized peak I_Na_ resistance to 3 mM lidocaine. *P* = 0.86; *t* test. (**J**) Persistent I_Na_ normalized to peak current. One-way ANOVA, *P* < 0.001; ** *P* < 0.01; *** *P* < 0.001. Mean ± SEM.

The F1759A-Na_V_1.5/reverse tetracycline-controlled transactivator protein mice (10) were generated by crossing mice with FLAG-tagged F1759A human *SCN5A* (Na_V_1.5) fused to a modified murine α–myosin heavy chain, tetracycline-inducible promoter vector (24) and cardiac-specific expression of reverse tetracycline-controlled transactivator protein (rtTA) (Figure 1A) (25). The mice with both F1759A-Na_V_1.5 and rtTA transgenes have a low level of F1759A-Na_V_1.5 channel expression in the absence of doxycycline (10, 15), probably due to a low basal binding of rtTA protein to the *Tet* operator sequences (“leak”) (25). The F1759A substitution markedly diminishes use-dependent lidocaine block (26), enabling the differentiation of the electrophysiological profiles of endogenous and mutant transgenic Na^+^ channels in cardiomyocytes. The mean peak Na_V_1.5 current in isolated atrial cells, determined with 5 mM Na^+^ rather than physiological Na^+^ in the extracellular solution to mitigate voltage clamp errors, was not significantly different in atrial cardiomyocytes isolated from F1759A-Na_V_1.5 mice and nontransgenic mice (Figure 1, B, C, and H). As we previously reported (10, 15), the F1759A mutation prevented complete inactivation of Na_V_1.5, thereby increasing persistent Na^+^ current in atrial cardiomyocytes isolated from F1759A mice (Figure 1, B, C, and J). The increased persistent Na^+^ current in the F1759A-Na_V_1.5 atrial cardiomyocytes led to an elevation in the intracellular Na^+^ concentration ([Na^+^]_i_) of quiescent and field-stimulated atrial cardiomyocytes compared with nontransgenic mice (Figure 1D). Due to the expression of F1759A-Na_V_1.5 channels and the increased [Na^+^]_i_, the mice developed structural alterations, including atrial and ventricular enlargement, myofibril disarray, fibrosis, mitochondrial necrosis, and electrophysiological dysfunctions, leading to spontaneous and prolonged episodes of AF (10).

We crossbred these mice with mice expressing human catalase in mitochondria (mCAT) driven by a CMV enhancer/chicken β-actin promoter (27, 28), yielding triple-transgenic mice (Figure 1E). Using the triple-transgenic mice and littermate controls of double-transgenic mice expressing only F1759A-Na_V_1.5 and rtTA transgenes, we sought to determine whether reducing mitochondrial ROS by expression of mCAT would attenuate the structural remodeling and arrhythmogenesis associated with increased persistent Na^+^ current. Similar to F1759A-Na_V_1.5 transgenic mice, the mice with both F1759A-Na_V_1.5 and mCAT transgenes demonstrated a low level of F1759A-Na_V_1.5 channel expression in the absence of doxycycline, and expression of mCAT did not affect the transgenic expression of the F1759A-Na_V_1.5 channels (Figure 1F). The mean peak Na_V_1.5 current in isolated atrial cells was not significantly different in F1759A mice and F1759A-mCAT mice (Figure 1, C, G, and H). Furthermore, consistent with protein expression levels, the peak transgenic Na^+^ current, assessed by superfusion of 3 mM lidocaine, which inhibits wild-type nontransgenic Na^+^ current (10), was equivalent in F1759A-Na_V_1.5 and F1759-Na_V_1.5-mCAT atrial cardiomyocytes (Figure 1I). Since mCAT expression reduces oxidative stress, we determined whether the expression and function of F1759A-Na_V_1.5 channels were affected by altering the oxidative status within cardiomyocytes. Na_V_1.5 channels contain methionine residues that can impair open-state inactivation when oxidized (29). We reasoned that the F1759A mutation rather than the oxidative status of the channel was responsible for the increased persistent Na^+^ currents that we previously observed (10, 15). Expression of mCAT did not affect the amount of persistent Na^+^ current in atrial cardiomyocytes (Figure 1, G and J), likely because the F1759A mutation causes an intrinsic defect in channel inactivation.

**Figure 2:**
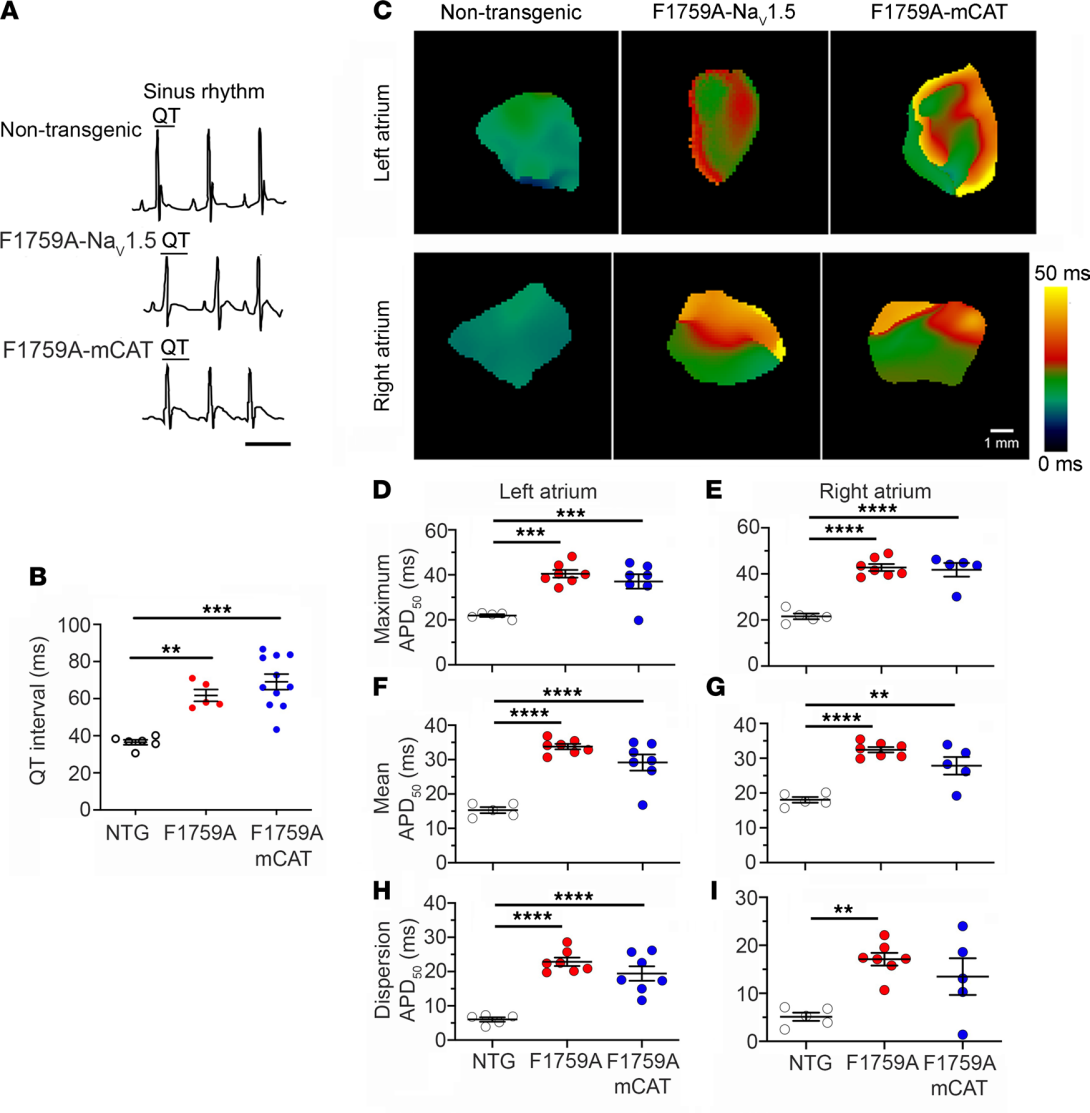
Expression of mitochondrial catalase does not attenuate F1759A-Na_V_1.5–induced prolongation of the action potential duration. (**A**) Representative limb-lead surface electrocardiograms of isoflurane-anesthetized nontransgenic control mice, F1759A, and F1759A-mCAT mice in normal sinus rhythm. Scale bar: 100 ms. (**B**) Graph of QT intervals. Mean ± SEM. One-way ANOVA, *P* < 0.0001; ** *P* < 0.01; *** *P* < 0.001 by Tukey’s multiple-comparison test. (**C**) Representative optical APD_50_ maps of right and left atria of nontransgenic, F1759A, and F1759-mCAT mice. APD maps (pacing at 10 Hz) for F1759A-Na_V_1.5 mice were obtained after hyperkalemia-induced conversion to sinus rhythm. Scale bar: 1 mm. (**D** and **E**) Graph showing maximal APD_50_ in left and right atria of NTG, F1759A-NaV1.5, and F1759A-mCAT mice. Mean ± SEM. One-way ANOVA, *P* < 0.001 for left atrium, *P* < 0.0001 for right atrium. *** *P* < 0.001; **** *P* < 0.0001 by Tukey’s multiple-comparison test. (**F** and **G**) Graphs of mean APD_50_. Mean ± SEM. *P* < 0.0001 by 1-way ANOVA; ** *P*< 0.01; **** *P* < 0.0001 by Tukey’s multiple-comparison test. (**H** and **I**) Graphs of APD_50_ dispersion. Mean ± SEM, 1-way ANOVA, *P* < 0.0001 for left atrium, *P* < 0.01 for right atrium. ** *P* <0.01; **** *P* < 0.0001 by Tukey’s multiple-comparison test.

We measured the QT interval, a marker of ventricular repolarization, in sinus rhythm. Although the F1759A transgenic mice have paroxysmal AF, they have substantial periods of stable sinus rhythm enabling these measurements. Consistent with increased persistent current, the QT interval increased in the F1759A mice and the F1759A-mCAT mice compared with nontransgenic control mice (Figure 2, A and B). Epicardial surface optical voltage mapping of the anterior surface of Langendorff-perfused control, F1759A, or F1759-mCAT hearts was used to assess atrial repolarization (Figure 2C). As we have done previously (15), the Langendorff apparatus–mounted hearts were perfused with a hyperkalemic solution to terminate the arrhythmias in order to elucidate the underlying electrophysiological substrate. Thereafter, a normokalemic solution was infused, and the atrial APD was measured by pacing the atria at 10 Hz. The maximal and mean APD were increased by about 2-fold in both right and left atria of the F1759A and F1759-mCAT mice compared with control mice (Figure 2, D–G). Consistently, we observed APD heterogeneity in both left and right atria — demonstrated by the nonuniformity in APD maps (Figure 2C) and the dispersion of APD, assessed by the difference between greatest and least APD (Figure 2, H and I). To summarize, coexpression of mCAT had no effect on the repolarization of the action potential of the atria and ventricle, implying that the mutation F1759A itself rather than posttranslational modifications of the transgenic Na_V_1.5 channels is responsible for the persistent Na^+^ current and prolonged repolarization in these mice. The unabated increased persistent Na^+^ current in the presence of mCAT expression enabled us to explore the downstream role of ROS in mediating persistent Na^+^ current-induced structural and electrophysiological remodeling in the atria.

### Mitochondrial expression of catalase attenuates ROS in F1759A-Na_V_1.5 expressing mice.

**Figure 3:**
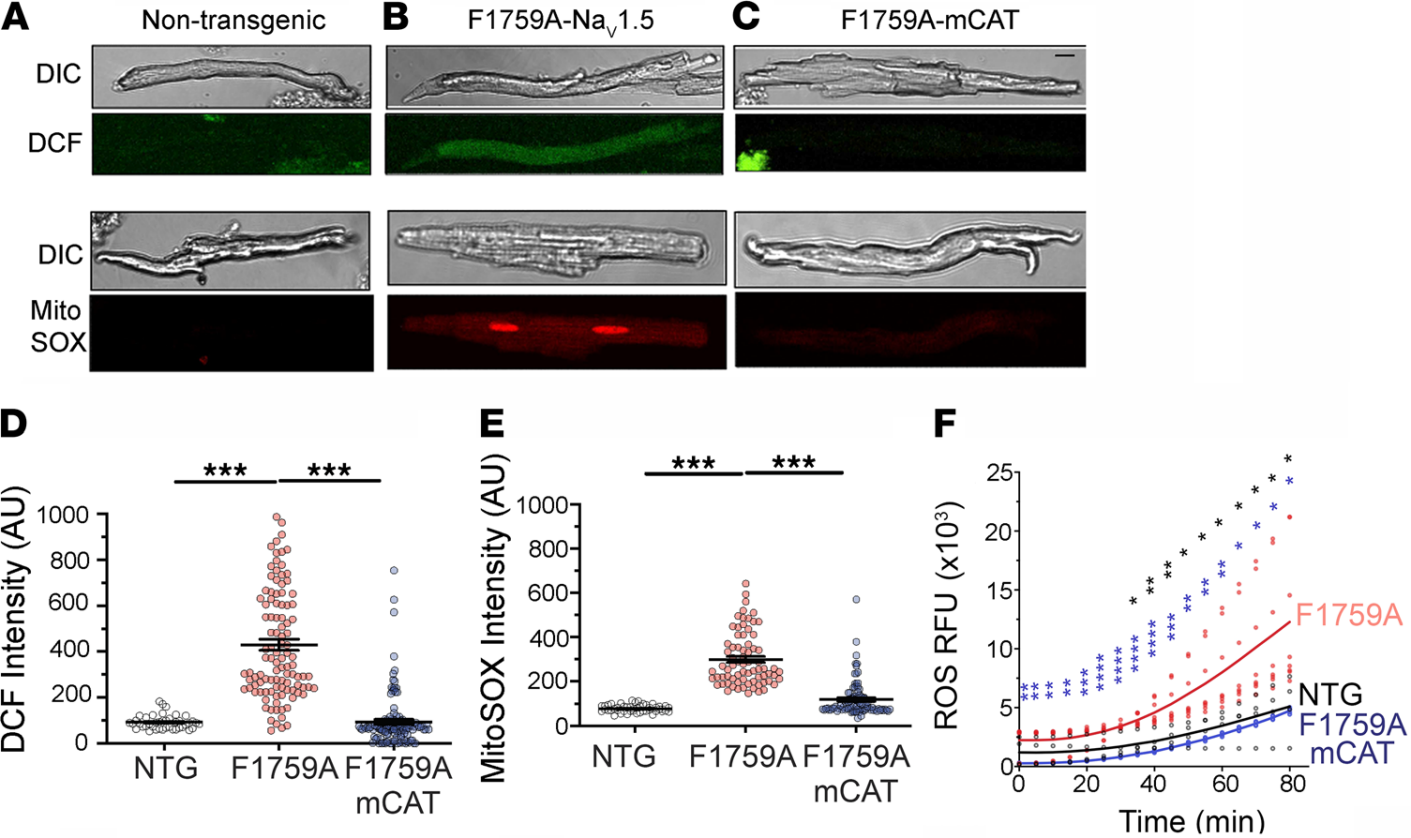
Persistent Na^+^ current causes mitochondrial dysfunction, which is attenuated by expression of mitochondrial catalase. (**A**–**C**) Representative differential interference contrast (DIC) microscopy and fluorescent images of atrial cardiomyocytes stained with either 2′, 7′–dichlorofluorescein (DCF) or MitoSOX red. Scale bar: 15 μm. (**D**) Graphs of DCF fluorescence in arbitrary units (AU). From left to right *n* = 42, 106, and 122 atrial cardiomyocytes from *n* = 5 NTG, 5 F1759A, and 8 F1759A mCAT, respectively. *P* < 0.0001 by 1-way ANOVA; *** *P* < 0.0001 by Tukey’s multiple-comparison test. (**E**) Graph of MitoSOX red fluorescence in AU. From left to right, *n* = 47, 73, and 93 of atrial cardiomyocytes from *n* = 5 NTG, 5 F1759A, and 8 F1759A mCAT, respectively. *P* < 0.0001 by 1-way ANOVA, *** *P* < 0.0001 by Tukey’s multiple-comparison test. (**F**) ROS production in relative fluorescence units (RFU) from isolated cardiac mitochondria in nontransgenic, F1759A, and F1759A-mCAT mice. *n* = 4, 8, and 4 mice, respectively. *P* = 0.0002 by 2-way ANOVA; * *P* < 0.05, ** *P* < 0.01, *** *P* < 0.001, **** *P* < 0.0001 by Tukey’s multiple-comparison test. Black asterisks signify tests for NTG vs. F1759A; blue asterisks, F1759A vs. F1759A-mCAT.

Postoperative, paroxysmal, and long-standing AF are associated with increased atrial ROS (17, 30). One important source of ROS is mitochondria, which during AF not only undergo severe structural and morphological alterations (swelling and disturbance of cristae structure) but also have impaired function (31, 32). We isolated atrial cardiomyocytes from nontransgenic control, F1759A, and F1759A-mCAT mice. We used the ROS indicator dihydrodichlorofluorescein diacetate (H_2_DCF-DA) as a measure of general oxidative stress in live cells, and MitoSOX Red, a mitochondrial superoxide indicator. Isolated cardiomyocytes from F1759A transgenic mice had significantly increased DCF and MitoSOX Red signals compared with control (Figure 3, A, B, D, and E). In contrast, expression of mCAT attenuated the increase of both cellular and mitochondrial ROS (Figure 3, C–E). We also measured ROS production in mitochondria isolated from the atria and ventricles of these mice. Expression of catalase in mitochondria markedly attenuated the production of ROS (Figure 3F). Taken together, we can conclude that reducing mitochondrial ROS by expression of catalase in the mitochondria also suppresses cellular ROS in the F1759A mice.

**Figure 4:**
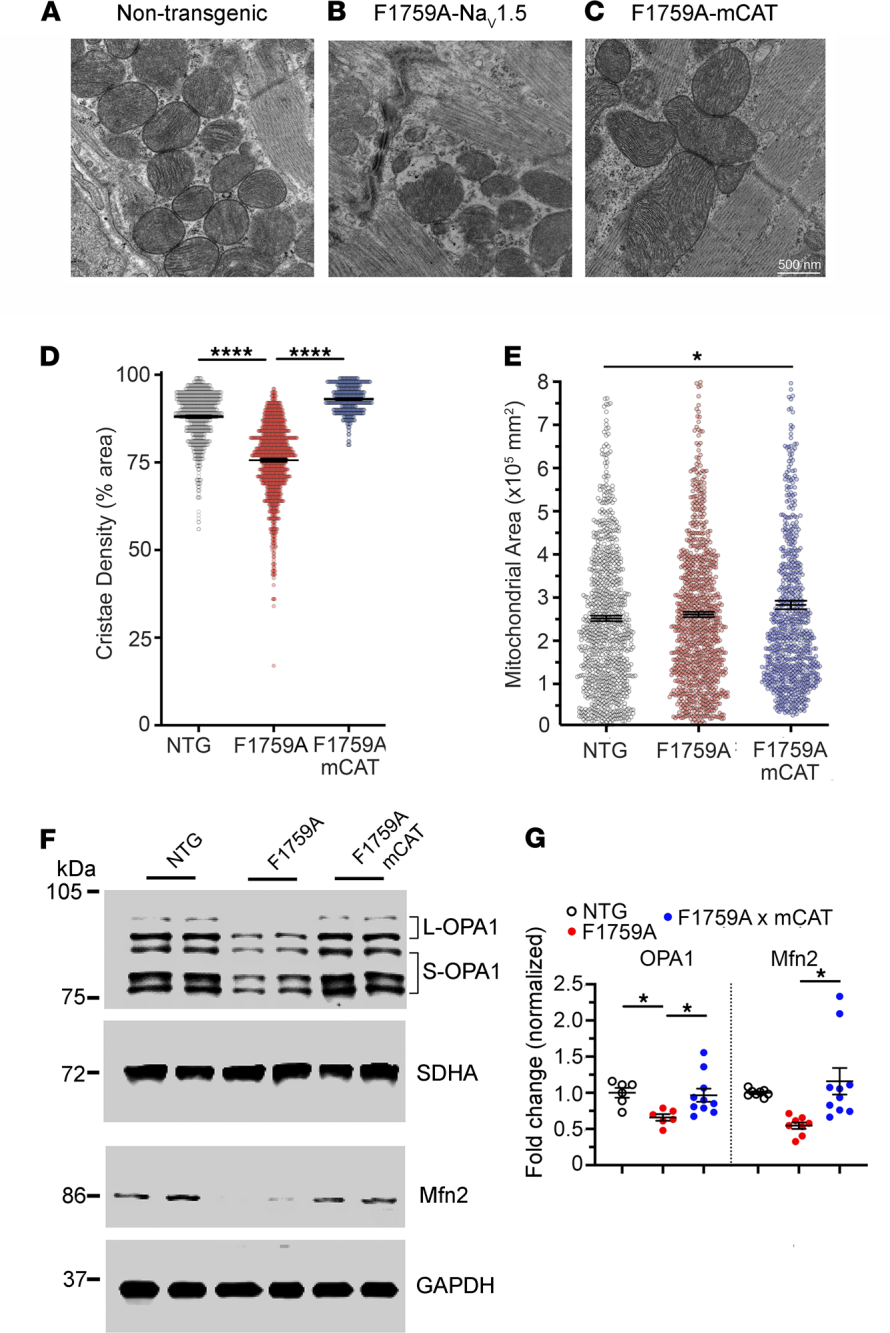
Persistent Na^+^ current induces changes in mitochondria cristae density and area. (**A**–**C**) Representative transmission electron microscopy images from atrial slices from control, F1759A, and F1759A-mCAT mice (*n* = 2 mice in each group). (**D**) Graph of cristae density. Mean ± SEM. *n* = 873, 963, and 756 mitochondria, respectively. *P* < 0.0001 by 1-way ANOVA; **** *P* < 0.0001 by Tukey’s multiple-comparison test. (**E**) Graph of mitochondria area. Mean ± SEM. *n* = 873, 963, and 756 mitochondria, respectively. *P* < 0.05 by 1-way ANOVA; * *P* < 0.05 (NTG vs. F1759-mCAT). (**F**) Representative immunoblots of OPA1 isoforms and Mfn2 from nontransgenic, F1759A, and F1759A-mCAT mouse hearts. Anti-SDHA and anti-GAPDH blots are used as loading controls. See complete unedited blots in the supplemental material. (**G**) Graphs of normalized fold change for OPA1 and Mfn2. *P* < 0.01 by 1-way ANOVA. * *P* < 0.05 by Tukey’s multiple-comparison test.

Whereas normal mitochondria use oxidative phosphorylation to generate ATP, damaged mitochondria become a production site for ROS (33). Excessive ROS production can facilitate autophagy and apoptotic stress. In atrial cardiomyocytes, increased persistent Na^+^ current was sufficient to initiate mitochondrial injury including circular and swollen mitochondria, ruptured outer membranes, and reduced density of cristae (Figure 4, B and D), which was not observed in nontransgenic littermate control mice (Figure 4A). In atrial cardiomyocytes of mice with expression of both F1759A-Na_V_1.5 and mCAT (Figure 4C), the mitochondria were larger, with increased cristae density than the mitochondria observed in both nontransgenic control mice and F1759A mice (Figure 4, D and E). Mitochondrial membrane fusion is catalyzed by the mitofusins (Mfn1/2) and Opa1, which are members of the dynamin family of large GTPases (34, 35). We found that in the atria of F1759A mice, the expression of Opa1 and Mfn1 was reduced compared with control mice (Figure 4, F and G). Thus, persistent Na^+^ current in the atria leads to mitochondrial dysmorphology and increased ROS, which can be attenuated by expression of catalase in mitochondria.

### Mitochondrial expression of catalase reduces structural remodeling in atria.

**Figure 5:**
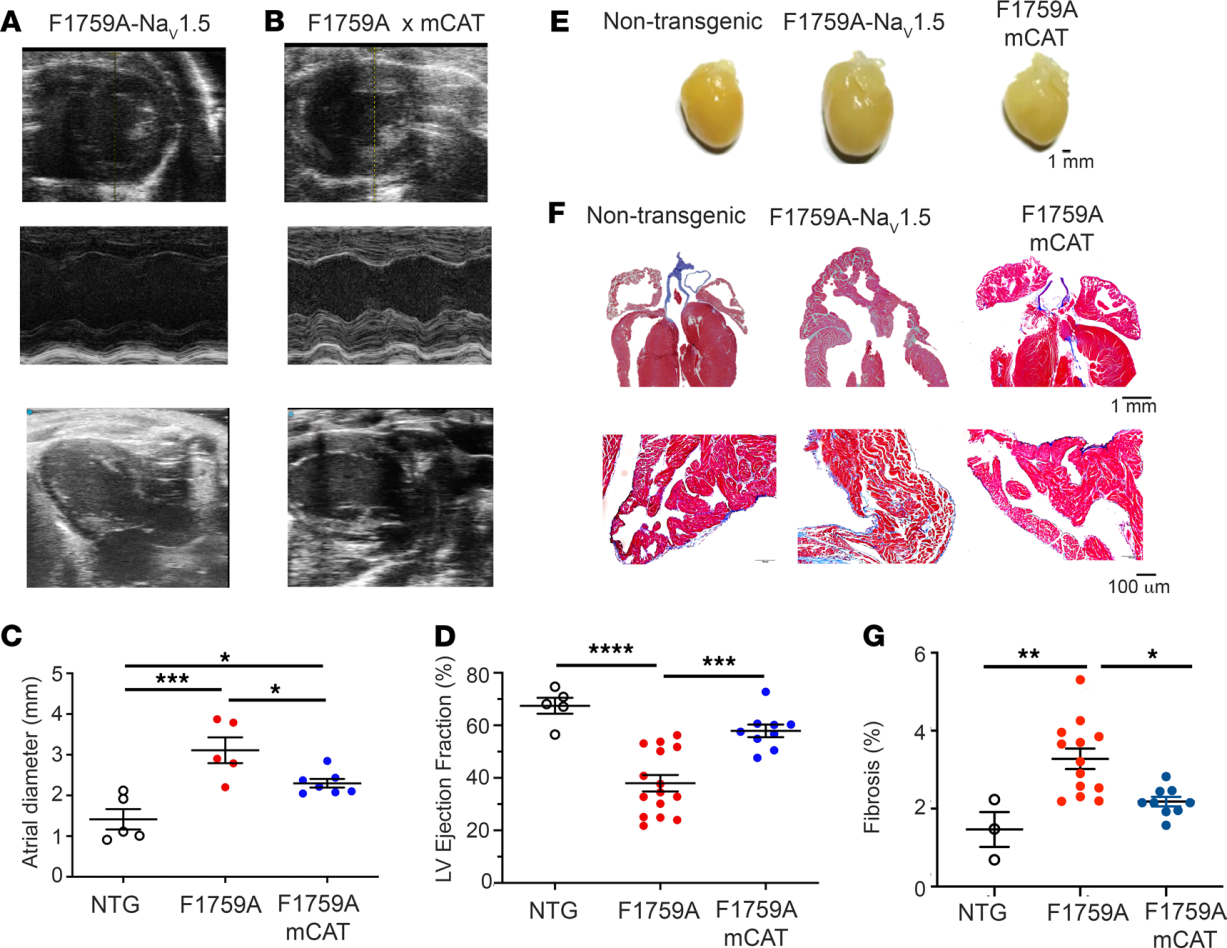
Expression of mCAT attenuates persistent Na^+^ current-induced cardiac structural remodeling. (**A** and **B**) Representative transthoracic echocardiography images (upper, short axis; middle, M-mode; lower, long axis) from F1759A and F1759A-mCAT mice. (**C**) Graph of left atrial diameter derived from echocardiographic studies. Mean + SEM. *P* < 0.001 by 1-way ANOVA. * *P* < 0.05; *** *P* < 0.001 by Tukey’s multiple-comparison test. (**D**) Graph of LV ejection fraction. Mean ± SEM. *P* < 0.0001 by 1-way ANOVA. *** *P* < 0.001, **** *P* < 0.0001 by Tukey’s multiple-comparison test. (**E**) Representative photographs of nontransgenic, F1759A, and F1759A-mCAT hearts at 4 months after birth. (**F**) Representative images of Masson’s trichome stain of hearts (upper) and of left atrium (lower) showing increased fibrosis in F1759A compared with nontransgenic and F1759-mCAT mice. (**G**) Graph quantifying atrial fibrosis. Data are presented as mean ± SEM. *P* < 0.01 by 1-way ANOVA. * *P* < 0.05, ** *P* < 0.01 by Tukey’s multiple-comparison test.

Elevated [Na^+^]_i_, caused by increased persistent Na^+^ current, is a hallmark of both animal models and human heart failure (36–38). Previously (10), we showed that increased persistent Na^+^ current is sufficient to cause progressive dilation of both atria and ventricles, assessed by echocardiography (Figure 5, A–E); reduced left ventricular function (Figure 5D); and increased fibrosis (Figure 5, F and G). The mechanism of modestly reduced LV function was likely multifactorial, due to both intrinsic contractility defects and secondarily due to a tachycardia-induced cardiomyopathy and/or the difficulty in assessing LV function in the setting of both AF and ventricular arrhythmias. The mechanism by which persistent Na^+^ current causes cardiac chamber dilatation and fibrosis was not clear. Increased mitochondrial ROS is known to drive detrimental structural remodeling in a variety of cardiac diseases. For instance, expression of catalase in mitochondria reduces autophagy and hypertrophy in angiotensin II–treated mouse hearts (39, 40) and improves contractile dysfunction in mice with metabolic syndrome caused by high-fat, high-sucrose diet (41). Similarly, we found that mCAT expression caused a reduction in both atria and ventricle size and fibrosis compared with those mice only expressing F1759A-Na_V_1.5 (Figure 5, B–G). Cardiac function was also improved in the mCAT-expressing F1759A transgenic mice compared with F1759A mice (Figure 5D), which could be the result of a reduction in arrhythmias, as we show below, or improvement in intrinsic contractile function. These findings offer direct evidence for the central role of mitochondrial ROS in driving the structural remodeling caused by increased persistent Na^+^ current.

### Mitochondrial expression of catalase reduces atrial arrhythmias.

**Figure 6:**
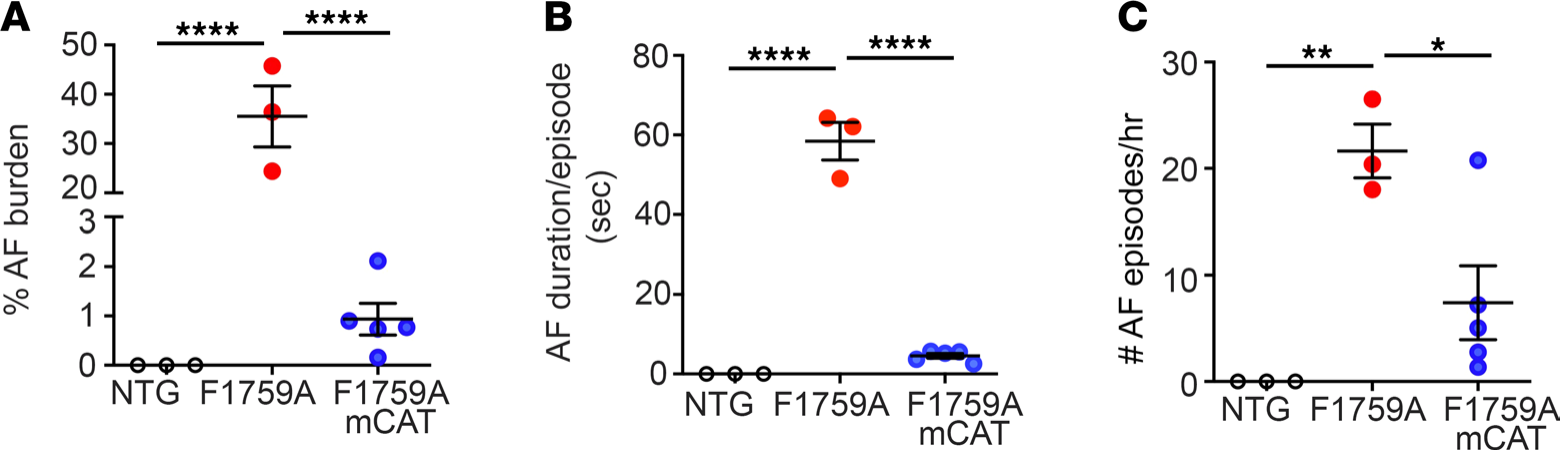
Reducing mitochondrial ROS attenuates persistent Na^+^ current-induced spontaneous AF. (**A**) Graph showing percentage of AF during 20-hour telemetry recordings in nontransgenic control mice, F1759A mice, and F1759A-mCAT mice. *P* < 0.0001 by 1-way ANOVA. **** *P* < 0.0001 by Tukey’s multiple-comparison test. (**B**) Graph of mean (± SEM) duration of AF in seconds. *P* < 0.0001 by 1-way ANOVA. **** *P* < 0.0001 by Tukey’s multiple-comparison test. (**C**) Graph of mean number of AF episodes/hour in each mouse. *P* < 0.01 by 1-way ANOVA. * *P* < 0.05; ** *P* < 0.01 by Tukey’s multiple-comparison test.

We implanted subcutaneous electrocardiography (ECG) telemeters to determine the AF burden in the F1759A-Na_V_1.5 and F1759A-mCAT mice. Spontaneous AF was never observed in nontransgenic control mice (Figure 6A). Spontaneous AF in nonanesthetized mice was detected in the 3 F1759A mice with implantable ECG telemeters, consistent with our prior studies (10, 15), with an average AF burden of about 35%, average duration of AF of 58.4 seconds, and average number of AF episodes per hour of 21.6 (Figure 6, A–C), implying that the mice had a substantial burden of paroxysmal AF. Coexpression of mCAT, however, markedly reduced the AF burden to an average of less than 1%, an average duration of 4.5 seconds, and an average number of episodes per hour of 7.4. Overall, attenuating mitochondrial ROS reduced paroxysmal AF burden by more than 95%.

**Figure 7:**
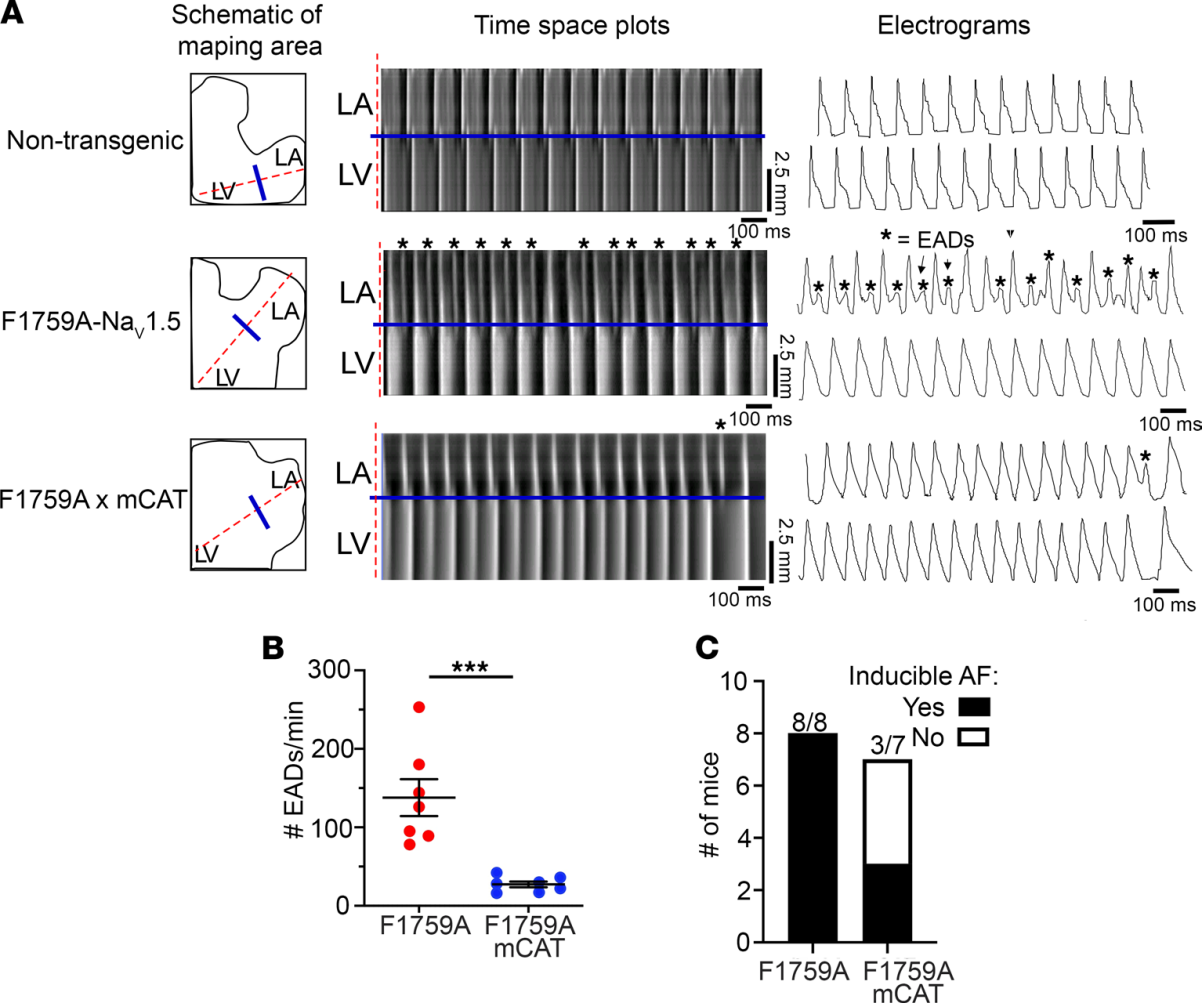
Reducing mitochondrial ROS attenuates EADs and inducible AF. (**A**) Time-space plots of left atrium (LA) and left ventricle (LV) during 10 Hz atrial pacing. EADs are marked with asterisks. Single-pixel electrograms showing EADs. (**B**) Graph of EADs per minute. *** *P* < 0.001 by unpaired 2-tailed *t* test. (**C**) Bar graph depicting number of mice subjected to electrophysiological testing for AF inducibility and the number of mice with AF. *P* = 0.03 by Fisher’s exact test.

We acquired optical voltage maps concurrently with burst pacing of the atria to induce AF. Consistent with the hypothesis that increased APD causes EADs that can trigger arrhythmias, we observed, using optically acquired voltage maps and time-space plots, pacing-induced phase 3 EADs in the F1759A mice, which were never observed in control, nontransgenic mice (Figure 7, A and B). In mice with expression of mCAT and F1759A-Na_V_1.5, the number of EADs diminished by 80% compared with those mice with expression of only F1759-Na_V_1.5. The reduction of EADs correlated with the reduced frequency of AF induction (Figure 7C): whereas AF was induced by 20 Hz pacing in 100% of F1759A mouse hearts (8 of 8), AF was induced in only 43% (3 of 7) of F1759A-mCAT mice hearts (*P* = 0.0256 by Fisher’s exact test). The reduction in AF inducibility is consistent with the decrease in frequency and duration of spontaneous AF (Figure 6, A–C).

**Figure 8:**
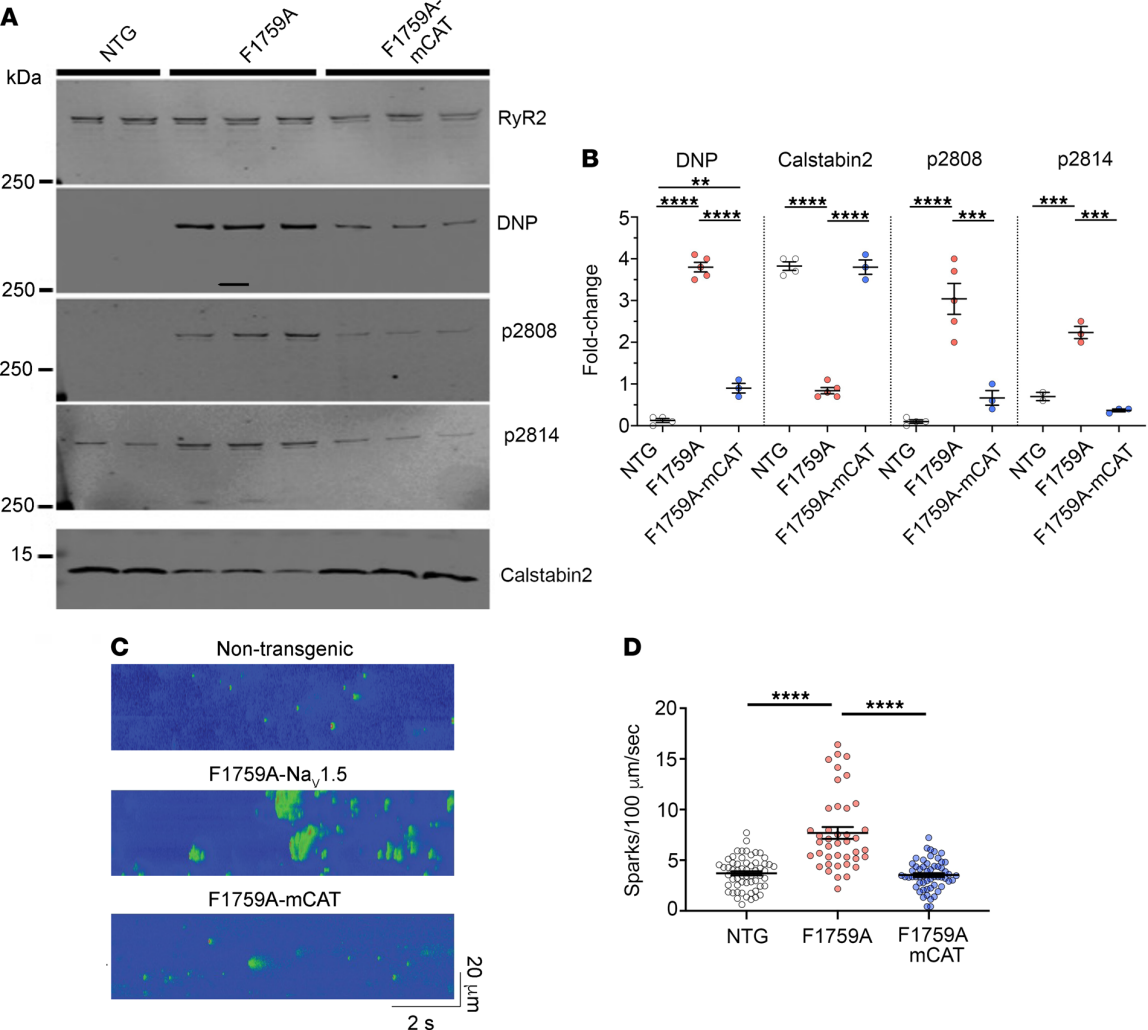
Persistent Na^+^ current induces RyR2 dysfunction, which is attenuated by expression of mitochondrial catalase. (**A**) Representative anti-RyR, anti-DNP, anti-Calstabin2, and anti–phospho-Ser2808 (p2808) and Ser2814 (p2814) antibody immunoblots of RyR2 immunoprecipitates from atrial tissue of nontransgenic, F1759A, and F1759A-mCAT mice. See complete unedited blots in the supplemental material. DNP, 2,4-dinitrophenol. (**B**) Graphs of quantification normalized to immunoprecipitated RyR2. *P* < 0.0001 by 1-way ANOVA for DNP, Calstabin2, and p2808, *P* < 0.001 for p2814. **** *P* < 0.0001, *** *P* < 0.001, ** *P* < 0.01 by Tukey’s multiple-comparison test. (**C** and **D**) Representative Ca^2+^ spark images and graph of Ca^2+^ spark frequencies from isolated cardiomyocytes from atria of nontransgenic controls, F1759A, and F1759A-mCAT mice. From left to right, *n* = 58, 41, and 56 atrial cardiomyocytes from 3 mice in each group. *P* < 0.0001 by 1-way ANOVA; **** *P* < 0.0001 by Tukey’s multiple-comparison test.

RyR2-mediated diastolic sarcoplasmic reticulum (SR) Ca^2+^ leak is associated with AF (8, 19, 42). The increased diastolic leak is caused by oxidation and phosphorylation of RyR2. We reasoned that RyR2 oxidation and SR Ca^2+^ leak could play important roles in enhancing AF in the F1759A-Na_V_1.5 mice and that reducing mitochondrial and cytosolic ROS could restore normal RyR2 function. Atrial RyR2 from F1759A-Na_V_1.5 mice exhibited increased oxidation and phosphorylation of the PKA (Ser2808) and calcium/calmodulin-dependent protein kinase II (CaMKII) (Ser2814) sites on RyR2, and depletion of RyR2-associated calstabin2, compared with wild-type control mice (Figure 8, A and B). To assess intracellular Ca^2+^ leak and its effects, we examined Ca^2+^ spark frequency. Ca^2+^ spark frequency was significantly increased (Figure 8, C and D) in atrial cardiomyocytes isolated from F1759A mice compared with control (Ca^2+^ sparks/100 μm/s: 3.7 ± 0.2 in WT vs. 7.7 ± 0.6 in F1759A, *P* < 0.0001). In contrast, coexpression of mCAT dramatically reduced RyR2 oxidation, decreased phosphorylation of RyR2 at Ser2808 and Ser2814, restored calstabin2 binding to RyR2 (Figure 8, A and B), and normalized Ca^2+^ spark frequency (Ca^2+^ sparks/100 μm/s: 3.5 ± 0.2) (Figure 8, C and D). Taken together, persistent Na^+^ current via increased mitochondrial ROS causes RyR2-mediated SR Ca^2+^ leak, likely contributing to the increased frequency of EADs and AF in the F1759A-Na_V_1.5 transgenic mice.

## Discussion

Enhanced Na^+^ influx can initiate and sustain the development of AF, as we show in a transgenic mouse model and as is observed in patients with gain-of-function *SCN5A* genetic variants (11, 12), and permanent AF (13). We demonstrate that increased persistent Na^+^ current in cardiomyocytes induces mitochondrial oxidative stress, which activates signaling pathways causing both electrophysiological and structural remodeling of the atria, leading to spontaneous AF in mice. Whereas increased persistent Na^+^ current can directly prolong the APD due to persistent cation influx, the other phenotypes that we observed, namely atrial enlargement and fibrosis, are indirect consequences of the increased persistent Na^+^ current in cardiomyocytes. We found that increased ROS is required for persistent Na^+^ current to impart these phenotypes. Many of the triggers of oxidative stress, such as age, diabetes, smoking, and inflammation, are associated with an increased risk of AF in humans (43–47). Previously, we showed that the perpetuation of AF in the F1759A-Na_V_1.5 mice required a heterogeneous increase of the APD (48). In this study, we show that the heterogeneous increase of the APD is not sufficient as the burden of AF was reduced by the suppression of oxidative stress in the mCAT-expressing mice.

It is possible that oxidative stress is both the consequence and cause of AF (30, 49). The mechanisms underlying increased ROS may differ at different stages of AF. Rac1 and NADPH oxidase activity and the protein level of NOX2 and p22phox were significantly increased in the left atrium of goats after 2 weeks of AF and in patients who developed postoperative AF (50). In the presence of long-standing AF or atrioventricular block, however, uncoupled nitric oxide synthase activity and mitochondrial oxidases accounted for the increase in ROS (50). Mitochondrial abnormalities are characteristic of atrial specimens from patients with AF (51). Swelling of the mitochondria is one of the first detectable responses to burst pacing–induced AF (52, 53). The high rate of electrical activity in AF leads to an increase in intracellular Na^+^ and Ca^2+^. Increased [Na^+^]_i_ can cause mitochondrial injury, potentially because elevation of [Na^+^]_i_ accelerates mitochondrial Ca^2+^ efflux and promotes ROS formation and oxidative stress (54, 55). In Ca^2+^-overloaded mitochondria, electron transport efficiency is diminished, leading to increased production of superoxide and decreased ATP synthesis (56, 57). In atrial tissues from patients with AF, oxidative stress and impaired mitochondrial structure and respiration were accompanied by activation of NF-κB signaling (58, 59), which can cause cardiomyocyte hypertrophy (60) and fibrosis (59). Blockade of Ca_V_1.2 current with verapamil prevented both the mitochondrial changes and the activation of NF-κB signaling, demonstrating that increased [Ca^2+^]_i_ contributes to oxidative stress during cardiac tachyarrhythmia (58). NF-κB is a central mediator of the priming signal of NACHT, LRR, and PYD domain containing protein 3 (NLRP3) inflammasome, which is activated in patients with AF (61). Inhibition of the NLRP3 has been shown to prevent spontaneous AF development in CREM transgenic mice.

Increased ROS modifies the activity of several ion channels in the atrium that may contribute to the development of AF. One mechanism is via enhanced activity of kinases, such as CaMKII. In patients with AF, there is an increase in atrial levels of methionine-oxidized CaMKII (19, 62–64), which is enzymatically active in the absence of Ca^2+^/CaM. CaMKII phosphorylates Na_V_1.5, Ca_V_1.2, and RyR2, increasing Na^+^ current, Ca^2+^ current, and SR Ca^2+^ release (29, 65–72). Another mechanism is oxidation and S-nitrosylation of ion channels. For instance, S-nitrosylation of Na_V_1.5 increases late Na^+^ current (73), although increased mitochondrial ROS has been reported to decrease peak Na^+^ current (74), which can also foster AF. S-nitrosylation and oxidation of RyR2 increases diastolic SR Ca^2+^ leak, which can promote after-depolarizations and AF (7, 19, 75). One relevant mechanism driving AF is diastolic SR Ca^2+^ leak, observed in atrial biopsies from humans with AF, and in several animal models with burst pacing–induced nonsustained AF (19, 42, 62, 76–83). The increased diastolic SR Ca^2+^ leak occurs with increased phosphorylation of RyR2 by PKA. Abnormal RyR2 function can also drive elevation of mitochondrial ROS, setting up a feed-forward, maladaptive signaling cascade that can perpetuate AF (19). We found that atrial RyR2 is oxidized, hyperphosphorylated at both PKA and CaMKII sites, and depleted of calstabin2 (FKBP12.6) in F1759A mice compared with nontransgenic controls. These changes in RyR2 cause diastolic SR Ca^2+^ leak. In atrial cells isolated from humans with permanent AF, there is a correlation between the extent of persistent Na^+^ current and diastolic SR Ca^2+^ leak, and in murine atrial cardiomyocytes, anemone toxin–induced persistent Na^+^ current caused increased SR Ca^2+^ leak (84). We found that attenuating the amount of mitochondrial ROS by expression of catalase markedly reduced RyR2 oxidation, RyR2-mediated Ca^2+^ sparks, and AF.

Our observations predict that attenuating mitochondrial ROS can prevent the development of persistent Na^+^ current-induced structural and electrophysiological remodeling that can sustain AF. Since the mitochondrial catalase is constitutively overexpressed throughout development and beyond, we cannot address whether expression of mitochondrial catalase can reverse the structural and electrophysiological abnormalities once these changes have developed. Reducing the oxidative stress by blocking the formation of ROS with antioxidants or blocking protein adduction with scavenger molecules has been tested for AF. In patients with post–cardiac bypass surgery, patients receiving oral ascorbate supplementation had significant reduction in postoperative AF from 34.9% in control subjects to 16.3% in the study cohort (85). In a small trial of patients with persistent AF treated with electrical cardioversion, AF recurrence was significantly reduced from 36% in the control group to 4.5% in the ascorbate-treated patients (86). In a meta-analysis, antioxidant treatment reduced the risk of postoperative AF, although subanalysis showed that only N-acetylcysteine and ascorbic acid had a beneficial effect (87). No significant benefit was observed in trials in which ascorbic acid was combined with other vitamins or in trials where vitamin E was employed. Notably, the trials were small, and since ascorbic acid is a weak antioxidant when given orally, it is unclear whether the reduction in AF was due to a direct antioxidant effect (87). Despite these tantalizing findings, modifying the atrial substrate using therapeutics with antiinflammatory, antifibrotic, and antioxidant properties, such as statins and polyunsaturated fatty acids, failed to demonstrate significant reductions in AF burden (88). Perhaps suboptimal antioxidants have been studied, and treatment of AF with more specific antioxidative therapies is possible.

In summary, this work demonstrates that increased persistent Na^+^ current in cardiomyocytes leads to elevated mitochondrial ROS, which drives both structural and electrophysiological abnormalities. Preventing the accumulation of ROS in cardiomyocytes attenuates atrial dilatation and fibrosis and after-depolarizations that cause the initiation and perpetuation of AF via enhanced diastolic SR Ca^2+^ release. Taken together, we identify the crucial role of increased mitochondrial ROS in mediating arrhythmias associated with increased persistent Na^+^ current.

## Methods

### General experimental approaches.

All experimental procedures and analysis were performed in a blinded fashion. No data points, samples, or mice were excluded from the study.

### Mouse model.

The transgenic constructs were generated by fusing human *SCN5A* cDNA (hH1) to the modified murine α-MHC tetracycline-inducible promoter vector (gift of Jeffrey Robbins and Jeffrey Molkentin, University of Cincinnati, Cincinnati, Ohio, USA) (24, 89) as described (10). The Na_V_1.5 cDNA was modified by inserting a 3X-FLAG epitope to the N-terminus and mutating F1759 to Ala. Transgenic mice with nontargeted insertion of this tetracycline-regulated cDNA (Figure 1A) were bred with cardiac-specific (α-MHC) codon-optimized rtTA mice (25) (obtained via the Mutant Mouse Resource and Research Center) to generate double-transgenic mice. These mice were crossbred with mCAT mice (stock number 016197, Jackson Laboratory) (28). F1759A-mCAT mice were positive for rtTA, F1759A, and mCAT transgenes, whereas F1759A-NaV1.5 mice were positive for rtTA and F1759A transgenes. Male and female mice, 6 weeks to 6 months of age, were used.

### Cellular electrophysiology.

Cardiomyocytes were isolated as described previously (90) from mice at least 12 weeks of age. Prior to euthanasia, surface ECG was obtained documenting AF in all F1759A mice. Experiments were performed at room temperature. Membrane currents from noncontracting rod-shaped cells with clear striations were measured by the whole-cell patch-clamp method using a MultiClamp 700B amplifier (Axon Instruments). The pipette resistance was 0.4–1.0 MΩ in order to minimize voltage clamp error. The liquid junction potential was corrected, series resistance was compensated, and the leak current was subtracted using a P/4 protocol. The intracellular (pipette) solution contained (in mM): 5 NaCl, 15 CsCl, 115 CsF, 10 HEPES, and 10 BAPTA, pH 7.4, titrated with CsOH. For persistent Na^+^ current determinations, the bath solution contained (in mM): 100 NaCl, 45 TEA-Cl, 10 HEPES, 1 MgCl_2_, 1 CaCl_2_, 5 glucose, pH 7.4, titrated with CsOH. The bath solution was then changed to reduce Na^+^ concentration to minimize Na^+^ current; in mM: 5mM NaCl, 140 TEA-Cl, 10 HEPES, 1 MgCl_2_, 1 CaCl_2_, 5 glucose, pH 7.4, titrated with CsOH. Lidocaine (3 mM) was superfused to determine the lidocaine-resistant current. Persistent current was evaluated by 190 ms depolarization from –100 mV to –30 mV in the absence and presence of ranolazine (Alomone). Digital subtraction of the time-averaged responses in the absence and presence of ranolazine yielded a small ranolazine-sensitive persistent Na^+^ current. The mean value of the last 10 ms of the 190 ms pulse was normalized to the peak Na^+^ current recorded using 5 mM Na^+^ in both intracellular and extracellular solutions.

### Measurements of [Na^+^]_i_.

Atrial [Na^+^]_i_ was measured as described previously (91, 92). Atrial myocytes were isolated using retrograde Langendorff perfusion (92, 93). Briefly, hearts were perfused with enzymatic solution containing 1 mg/mL collagenase type II, 0.06 mg/mL protease type XXIII, and 0.06 mg/mL trypsin for 5 minutes at 37°C. The atrial chambers were then separated, cut into small tissue strips, and subjected to an additional 10 minutes of enzymatic digestion at 37°C. Afterward cells were mechanically dissociated from the tissue by light agitation using a glass pipette. The cell suspension was then filtered through a nylon mesh (pore size 200 μm) and maintained in modified Tyrode’s solution (in mM: NaCl 130, KCl 5.4, CaCl_2_ 1.8, MgCl_2_ 0.5, NaH_2_PO_4_ 0.33, Glucose 5, HEPES 5, pH 7.4, adjusted with NaOH) supplemented with 2,3-butanedione monoxime (BDM, 10 mM). BDM was washed out 30 minutes prior to experiments by resuspension of atrial cells in normal Tyrode’s. Freshly isolated atrial myocytes were loaded with the Na^+^ indicator SBFI-AM (10 μM) for 60 minutes at room temperature. After 30 minute of de-esterification cells were seeded on a laminin-coated coverslip. Cells were mounted on an inverted microscope (Nikon Eclipse Ti2) connected to an EMCCD camera (Princeton Instruments Pro EM HS) and a rapid-switching illuminator with a 300 W xenon light source (DG5-plus, Sutter). Cells were continuously perfused with modified Tyrode’s solution (in mM: NaCl 130, KCl 5.4, CaCl_2_ 1.8, MgCl_2_ 0.5, NaH_2_PO_4_ 0.33, Glucose 5, HEPES 5, pH 7.4, adjusted with NaOH). Wide-field imaging was achieved with 40× objective (Nikon S Fluor, Oil, 1.30 NA) using 2 excitation wavelengths (340 nm and 380 nm) by fast switching scanning mirrors and narrow bandwidth excitation filters (340 nm ± 10 nm; 380 nm ± 10 nm). Emission light was collected at 510 ± 40 nm. Data were acquired using Nikon NIS-Elements software. SBFI fluorescence (F_340/380_) was collected after stable baseline recordings had been established for 5 minutes in quiescent cells. Atrial myocytes then underwent external field stimulation (2 ms, 20 V) at 1 Hz, and F_340/380_ was recorded after steady state was established. After completion of measurements, calibration of the F_340/380_ signal was performed in situ in each cell. The SBFI-loaded myocytes were exposed to 5 extracellular [Na^+^] ([Na^+^]_o_; 0–20 mM in 5 mM steps) in the presence of 10 μM gramicidin D and 100 μM strophanthidin. The solutions with various [Na^+^]_o_ were prepared by mixing, in different proportions, 2 solutions of equal ionic strength. One solution contained 145 mM Na^+^ (30 mM NaCl and 115 mM sodium gluconate) and no K^+^, while the other contained 145 mM K^+^ (30 mM KCl and 115 mM potassium gluconate) and was Na^+^ free. Both calibration buffers also contained 10 mM HEPES, 10 mM glucose, and 2 mM EGTA. The pH was adjusted to 7.2 with Tris base.

### Telemetry and ECG analysis.

Subcutaneous 3-lead ECGs of isoflurane-anesthetized mice were performed using EMKA ECG and recorded using Iox. QT intervals were measured manually using Ponemah 3 software. Telemetry devices (Data Sciences International) model (ETA-F10) were implanted in 4- to 6-month-old mice. Recordings were started 1 week after implantation. AF was defined as absence of P waves and irregular R-R intervals for more than 1 second. The electrocardiograms were analyzed blinded to genotype.

### Cardiomyocyte immunofluorescence and confocal microscopy.

Cardiomyocytes were incubated with DCF 25 μM or MitoSOX Red (5 μM) for 30 minutes in the dark (94). Excess DCF or MitoSOX Red was removed with 2 washes of BSA solution. DCF fluorescence was recorded at excitation/emission wavelengths 488/532 nm whereas MitoSOX Red was recorded at 525 (excitation) and 620 nm (emission). Confocal images were acquired using a Nikon A1R confocal microscope (Nikon Instruments Inc.) using a 40× objective. Images were analyzed for the fluorescence levels using Fiji (95).

### Immunoblots.

Cardiomyocytes were homogenized in a 1% Triton X-100 buffer containing (in mM): 50 Tris-HCl (pH7.4) 150 NaCl, 10 EDTA, 10 EGTA and protease inhibitors. The lysates were incubated on ice for 30 minutes and centrifuged at 21,912*g* at 4°C for 10 minutes, and then supernatants were collected. Proteins were size-fractionated on SDS-PAGE, transferred to nitrocellulose membranes, and probed with anti-FLAG (MilliporeSigma, catalog A8592), anti-Na_V_1.5 (Alomone, catalog ASC-005), and anti-tubulin (Santa Cruz Biotechnology, catalog sc-12462-R) antibodies. Detection and quantification were performed with a charge-coupled device camera (Carestream Imaging) and ImageQuant software, respectively.

RyR2 was immunoprecipitated from mouse atrial homogenates using a custom antibody (2.5 μg 5029 antibody) (96, 97). Immunoblots were developed for total RyR2, phosphorylation at Ser2808 and Ser2814, and calstabin2 (FKBP12.6) using custom-made antibodies, as previously described (97–100). To determine oxidation, the carbonyl groups in protein side chains in immunoprecipitants were derivatized (OxyBlot; MilliporeSigma) to 2,4-dinitrophenylhydrazone (DNP-hydrazone) by reaction with 2,4-dinitrophenylhydrazine and the DNP signal associated with RyR2 was determined using an anti-DNP antibody (MilliporeSigma, MAB 2223; 1:2000). Immunoblots were developed using the Odyssey Infrared Imaging System. All antibodies were validated prior to use.

For mitochondrial protein analysis, heart tissues were homogenized in 1 mL of 10 mM Tris-maleate buffer containing protease and phosphatase inhibitors (Roche Diagnostics). The homogenates were centrifuged 20 minutes at 8000*g*, and the supernatants were collected. Homogenates (50 μg) were size-fractionated on SDS-PAGE gels (6% for OPA1 and 4%–20% for the remaining proteins) and transferred onto nitrocellulose membranes. Immunoblots were probed with the following primary antibodies: OPA1 (BD Biosciences, mouse 612606, 1:1000), Mfn2 (Abcam ab56889, 1:1000), OXPHOS cocktail (Abcam, ab110413, 1:1000), GAPDH (MilliporeSigma, G9545, 1:1000), and SDHA (Abcam, ab137040, 1:1000). All immunoblots were developed using an Odyssey system (LI-COR Biosciences), with IR-labeled anti-mouse or anti-rabbit IgG (Abcam, 1:10000 dilution) secondary antibodies. Bands’ intensities were quantified using Image Studio Lite version 5.2.

### Echocardiography.

Transthoracic echocardiography was performed on anesthetized mice using a VisualSonics Vevo 2100 high-resolution imaging system with a 30 mHz imaging transducer. Left atrial diameter was measured at end-systole in parasternal long-axis view, and left ventricular end-diastolic dimension was measured in parasternal short-axis views and modified 4-chamber views. Left ventricular ejection fraction was measured in parasternal, modified 4-chamber, and short-axis views using Simpson’s biplane formula.

### Histology.

Excised hearts were placed in 10% formalin and cut in coronal sections. Every other slice (10 μm) was stained with hematoxylin-eosin and Masson’s trichrome staining for fibrosis. Each slide was reviewed and photographed under light microscopy 20×–400×. Fibrosis was evaluated as a ratio of total blue pixels: total myocardial area in Masson’s trichrome–stained slices, using cellSens imaging software (Olympus).

### Transmission electron microscopy.

The hearts were fixed with 2.5% glutaraldehyde in 0.1 M Sorenson’s buffer (pH 7.2), then postfixed with 1% OsO_4_ in Sorenson’s buffer for 1 hour. After dehydration, the hearts were embedded in Lx-112 (Ladd Research Industries, Inc.). Thin sections (60 nm) were cut on a PT-XL ultramicrotome. The sections were stained with uranyl acetate and lead citrate and examined under a JEOL JEM-1200 EXII electron microscope. Images were captured with an ORCA-HR digital camera (Hamamatsu) and recorded with an AMT Image Capture Engine. Image analysis performed using FIJI plugin.

### Optical mapping protocol.

Mice were injected with heparin prior to administration of isoflurane. Hearts were isolated and perfused via a Langendorff apparatus (Radnoti, LLC) with warm oxygenated Krebs-Henseleit buffer (pH 7.4; 95% O_2_, 5% CO_2_, 36–38°C) and were placed in a glass chamber in a Tyrode bath for superfusion. One AgCl wire was attached to the metal aortic cannula, and another AgCl wire was positioned near the surface of the heart to record an ECG. Blebbistatin (5–10 μM) was perfused to reduce motion, and Di-4-ANEPPS (100 μM) (both from Tocris Bioscience) was perfused to record optical membrane potentials. Hearts were uniformly shone with green excitation lasers (532 nm) to activate Di-4-ANEPPS. Emitted fluorescence was captured through a 580 nm pass filter using a complementary metal-oxide-semiconductor camera (MICAM Ultima, SciMedia). Movies were acquired at 1000 frames per second for a duration of 4–5 seconds, with 100 × 100 pixel resolution (0.095 mm per pixel). After the acquisition of optical movies during spontaneous rhythms, hearts were exposed to a hyperkalemic solution (8–14 mM K^+^), which terminated AF. Normokalemic solution was then infused. Susceptibility to pacing-induced AF was assessed by 3 attempts of burst pacing at twice the excitation threshold (Pulsar 6i, FHM) of the left atrium (20 Hz, amplitude 0.5–2.0 mA, 5 ms). All APD maps were generated after hyperkalemic conversion to sinus rhythm at 10 Hz pacing. For reinduction by pacing, AF was defined as a disorganized atrial arrhythmia for at least 1 second, which has been previously defined by others (19, 101).

Recorded optical movies were processed using custom software based on Precision Visuals — Workstation Analysis and Visualization Environment (Visual Numerics, Inc.) (102). The background fluorescence was subtracted from each frame, and spatial (5 × 5 pixels) and temporal (9 frames) conical convolution filters were used to increase signal-to-noise ratio. AF optical movies were spatially and temporarily filtered to reduce noise as AF usually occurs at a significantly lower amplitude. Movies recorded during pacing were averaged to improve signal-to-noise ratio. The optical APD was measured in each pixel at 50% repolarization level. Average APD, maximum APD, and APD dispersion (APD_max_ – APD_min_) were calculated for the whole atria for control, F1759A-Na_V_1.5, and F1759A-mCAT mice by drawing a custom 10 × 10 pixel area. In the APD maps, high APD gradients are regions where the difference between a long APD and a neighboring short APD within a 10 × 10 pixel area is greatest.

### Mitochondrial isolation and ROS production assay.

Isolation of mitochondria from mouse hearts was performed by differential centrifugation as previously described (103) with some modifications. Briefly, tissues were minced in buffer A (100 mM KCl, 5 mM MgSO_4_, 5 mM EDTA, 50 mM Tris-HCl, 0.5% BSA, pH 7.4) supplemented with 0.1 (wt/vol) of proteinase type XXIV (MilliporeSigma P8038) and incubated 5 minutes on ice. Tissue pellets were suspended in buffer B (buffer A + 1 mM of ATP, pH 7.4) and homogenized with a tissue grinder. After centrifugation at 8500*g* for 10 minutes at 4°C, the pellets were resuspended in buffer B, homogenized again with a tissue grinder, and centrifuged 10 minutes at 800*g*. The supernatants were collected, filtered, and centrifuged for 10 minutes at 9000*g* at 4°C. Pellets were resuspended in buffer B and centrifuged for 10 minutes at 9000*g* at 4°C. Mitochondrial pellets were resuspended in 100 μL of buffer C (100 mM KCl, 10 mM MOPS, pH 7.4). Mitochondrial protein concentration was determined using the bicinchoninic acid assay (Thermo Fisher Scientific) and subsequently diluted to a working concentration of 10 mg/mL in buffer C. Mitochondrial fractions (50 μg) were incubated in VO_2_ buffer (250 mM sucrose, 50 mM KCL, 25 mM Tris-HCL, and 10 mM K_2_HPO_4_, pH 7.4) in a 96-well black plate at 37°C. ROS production was assessed at 37°C for 80 minutes by adding 50 μM H_2_DCF-DA (Invitrogen) as previously described (104). ROS production is proportional to fluorescence emission monitored at ex-485/em-528 nm with a microplate fluorimeter. Microplate data were compiled and analyzed using i-control microplate reader software (Tecan), and results were expressed as arbitrary fluorescence units.

### Calcium sparks.

Atrial myocytes were enzymatically dissociated from the hearts of control, F1759A, and F1759A-mCAT mice using the Langendorff retrograde perfusion method. Cardiomyocytes were loaded with fluo-4 (5 μM for 10 minutes) in modified Tyrode’s solution containing 1 mmol/L Ca^2+^. Calcium sparks and line scan images were recorded with a Leica SP2 confocal microscope equipped with a 63× and 1.4× NA objective. Spark analysis was obtained using SparkMaster ImageJ plugin (NIH).

### Statistics.

Group data are presented as mean ± SEM. Statistical comparisons between the groups were tested using an unpaired *t* test and 1- or 2-way ANOVA tests for multiple comparisons. Values of *P* < 0.05 were considered statistically significant. All statistical analyses were performed with either GraphPad Prism 8 or OriginLab.

### Study approval.

The Institutional Animal Care and Use Committees at Columbia University and the University of Maryland approved all animal experiments.

## Author contributions

SOM, ARM, and EYW designed the study. UMRA, HD, BXC, ANK, YL, RJ, SRR, QY, ADD, SP, PD, HB, EM, CS, MG, HCJ, and EYW performed experiments. UMRA, HD, ANK, MG, HCJ, CWW, WJL, SOM, and EYW analyzed the data. UMRA, ARM, SOM, and EYW wrote the manuscript.

## Supplementary Material

Supplemental data

## Version 1 10/28/2021

In-Press Preview

## Version 2 12/08/2021

Electronic publication
