# Supplementary material for: Attenuating persistent sodium current–induced atrial myopathy and fibrillation by preventing mitochondrial oxidative stress
Source: JCI Insight. 2021 Oct 28;6(23):e147371. doi: 10.1172/jci.insight.147371 (PMC8675199; doi:10.1172/jci.insight.147371)
Supplement: Supplemental data [file jciinsight-6-147371-s038.pdf]

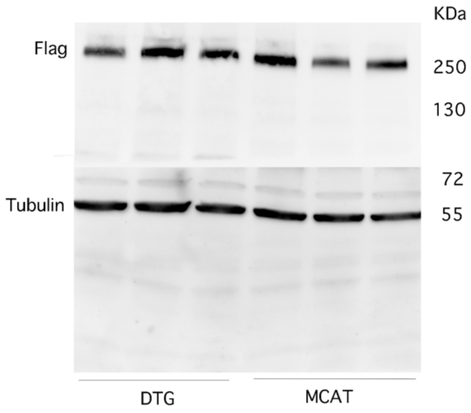


Unedited gel for Figure 1
(Nitrocellulose cut before blotting
top blotted with Anti-FLAG Ab
bottom blotted with anti-Tubulin Ab)

Unedited gels Figure 4

anti-OPA

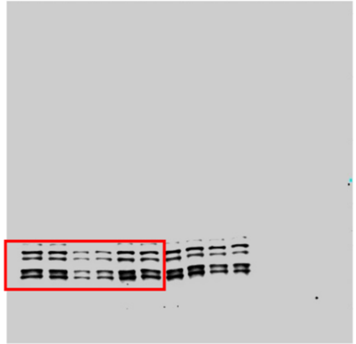

anti-Mfn2

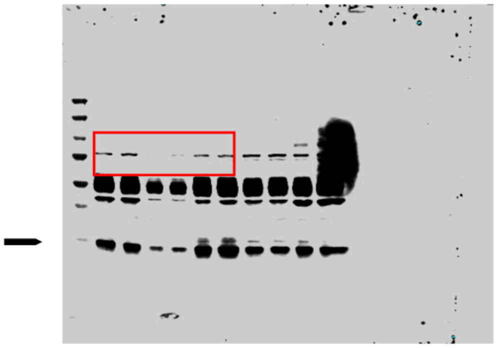

anti-SDHA

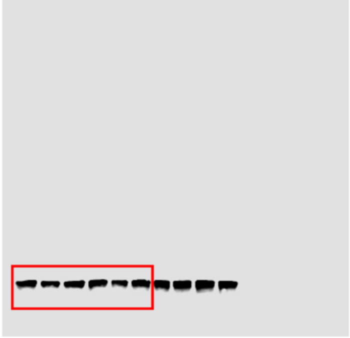

anti-GAPDH

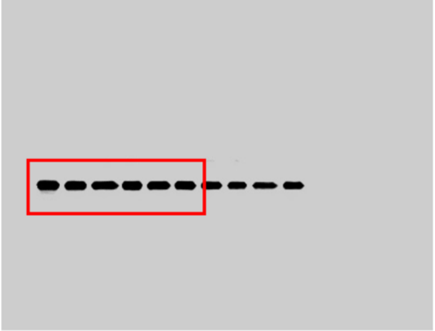

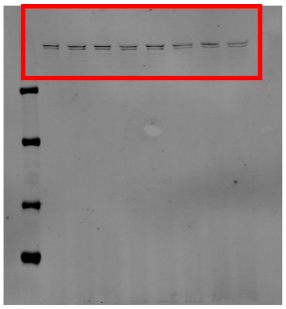

anti-RyR blot

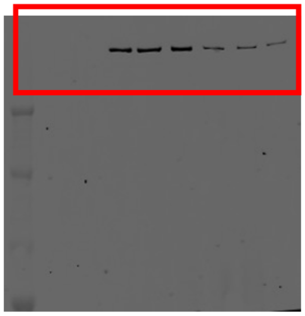

anti-DNP

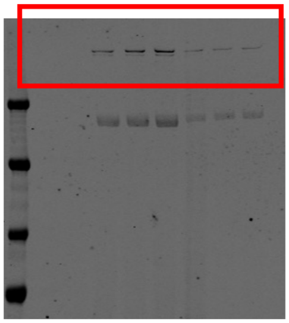

anti-p2808

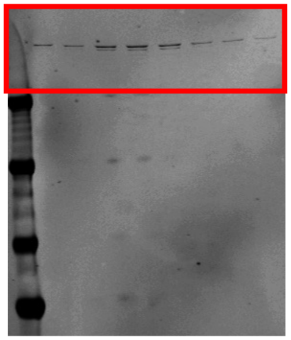

anti-p2814

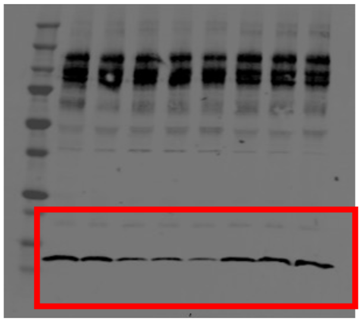

anti-calstabin

Unedited gels for Figure 8
